# Intravitreal bevacizumab associated with photodynamic therapy in a case of polypoidal choroidal vasculopathy associated with choroidal nevus: A case report

**DOI:** 10.1097/MD.0000000000009400

**Published:** 2017-12-29

**Authors:** Carlos M. Rangel, Eva Villota, Álvaro Fernández-Vega González, Ronald M. Sanchez-Avila

**Affiliations:** a Instituto Universitario Fernández-Vega, Fundación de Investigación Oftalmológica, Oviedo, Spain; b Fundación Oftalmológica de Santander (FOSCAL), Floridablanca, Colombia.

**Keywords:** antiangiogenic therapy, bevacizumab, choroid neoplasms, choroidal nevus, combined modality therapy, photodynamic therapy

## Abstract

**Rationale:**

Report the clinical findings and management of a case of polypoidal choroidal vasculopathy associated with choroidal nevus which received combination therapy.

**Patient concerns:**

Decreased visual acuity in a woman with polypoidal choroidal vasculopathy and choroidal nevus.

**Diagnoses:**

Polypoidal choroidal vasculopathy and choroidal nevus.

**Interventions:**

The initial visual acuity was 0.5. After the first treatment with photodynamic therapy, exudation and bleeding appeared around the lesion. After this, the patient received 3 doses of intravitreal bevacizumab.

**Outcomes:**

After treatment with combination therapy, visual acuity, clinical and imaging findings improved, with no recurrence of exudation and bleeding.

**Lessons:**

Intravitreal bevacizumab as an adjunctive treatment after photodynamic therapy is a good option for patients with polypoidal choroidal vasculopathy associated with choroidal nevus.

## 1 Introduction

Choroidal nevi are developmental tumors composed of benign melanocytes.^[1]^ They have the potential to cause visual loss and can resemble or transform into a choroidal melanoma. Rarely, subretinal neovascular membranes may be associated with choroidal nevi.^[2,3]^

Polypoidal choroidal vasculopathy (PCV), first described by Yannuzzi et al,^[4]^ consists of a branching vascular network with polypoidal lesions at its edge under the retinal pigment epithelium. Patients may experience recurrent subretinal hemorrhages, retinal pigment epithelium detachments, and hard exudates deposition.^[4]^

Association between choroidal nevus and PCV had been described, with positive results with therapy (laser photocoagulation and photodynamic therapy (PDT)).^[5–7]^

It was decided to report the case of a patient with PCV secondary to a choroidal nevus which was successfully treated intravitreal bevacizumab after 1 session of PDT.

## 2 Case report

**Figure 1:**
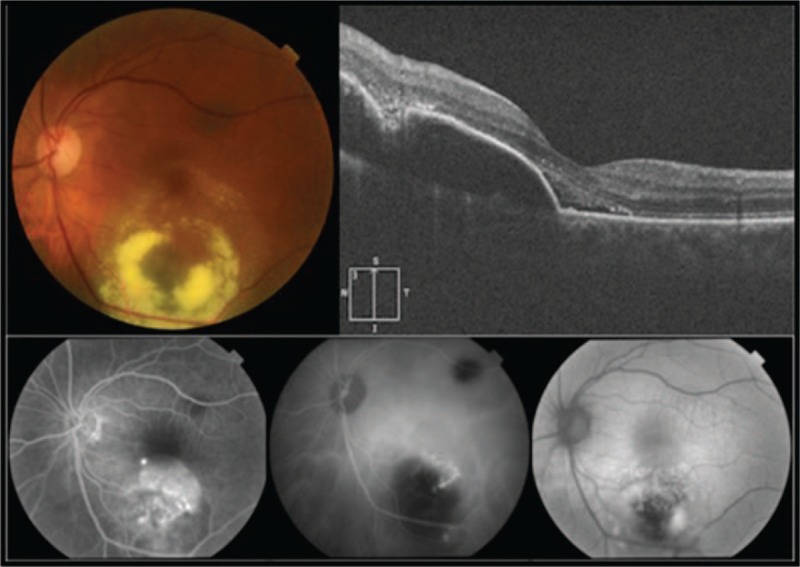
Fundus photograph, OCT, FA, ICG, and fundus autofluorescence of the left eye before PDT. Fundus photograph displaying 2 flat pigmented choroidal nevi: a subretinal elevated pigmented lesion in the inferior temporal vascular arcade surrounded by a ring of hard exudates that extends to the fovea. The other lesion in the superior temporal vascular arcade remains without exudation. Fluorescein angiography and ICG (bottom left and bottom middle) showing polypoidal lesions with minimal leakage in the late phases. Fundus autofluorescence (bottom right) did not show lipofuscin overlying the lesion. Optical coherence tomography scans (top right) show serous retinal pigment epithelium detachment, intra-retinal hyper-reflective foci, and associated shallow subfoveal fluid. FA = fluorescein angiography, ICG = indocyanine green angiography, OCT = optical coherence tomography, PDT = photodynamic therapy.

**Figure 2:**
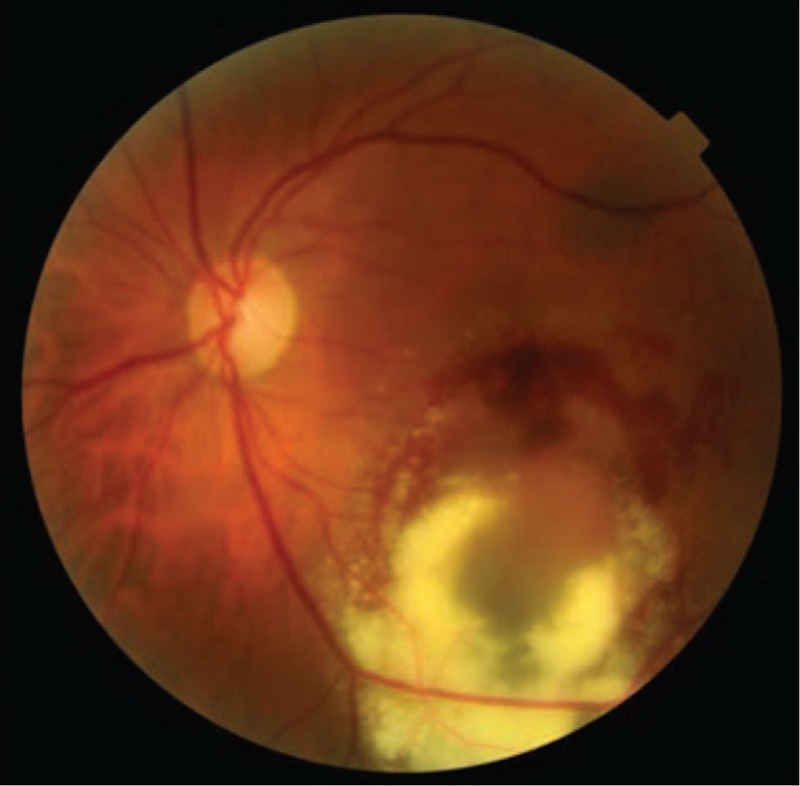
Fundus photograph 1 month after 1 session of PDT. Fundus photograph displaying increasing area of exudation, hard exudates, and bleeding. PDT = photodynamic therapy.

**Figure 3:**
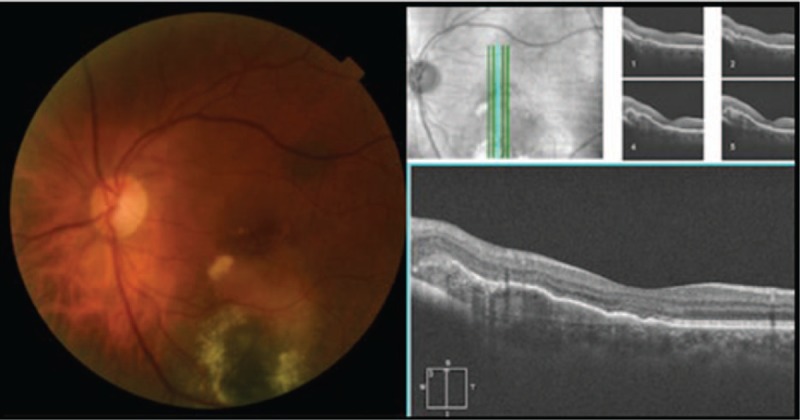
Fundus photograph and optical coherence tomography after 3 doses of intravitreal bevacizumab. Fundus photograph displaying 2 flat pigmented choroidal nevi: a subretinal elevated pigmented lesion in the inferior temporal vascular arcade surrounded by less amount of hard exudates without fovea compromise and no bleeding. The other lesion in the superior temporal vascular arcade remains without exudation. Optical coherence tomography showing no subretinal fluid, less intra-retinal hyper-reflective foci, and a hyper-reflective material accumulation under RPE. RPE = retinal pigmented epithelial.

**Figure 4:**
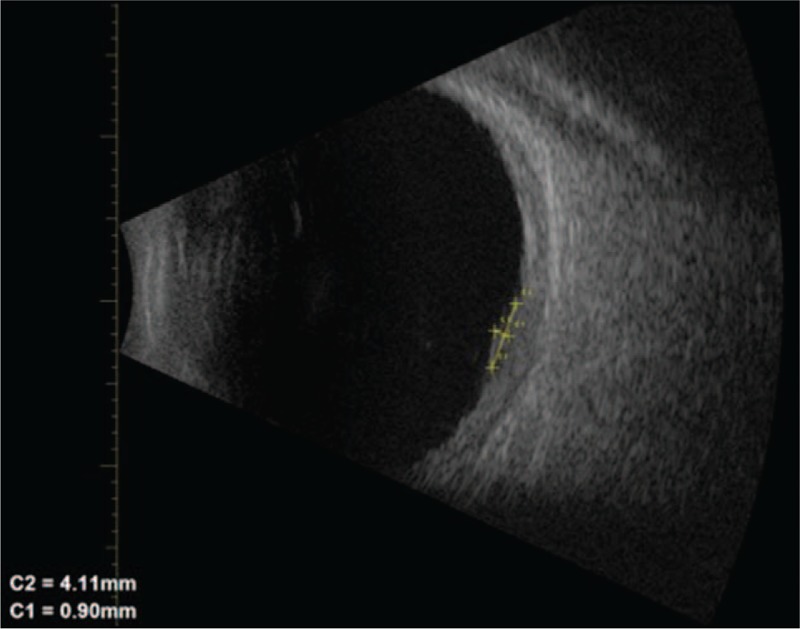
B-scan ultrasound performed when bleeding and exudation reduced significantly. Ultrasound B-scan showing a minimally elevated dome shaped, highly reflective choroidal lesion that measured 0.9 mm × 4.11 mm.

A 64-year-old woman present with a 3-day history of reduced vision in her left eye. Best corrected visual acuity (BCVA) was 1.0 in her right eye and 0.5 in her left eye. Slit-lamp examination shows no alteration in anterior segment in both eyes. Intraocular pressure was 16 mm Hg in both eyes. Fundus examination was normal in her right eye. Left eye showed a subretinal elevated pigmented lesion in the inferior temporal vascular arcade surrounded by a ring of hard exudates that extends to the fovea. Another subretinal flat pigmented lesion in the superior temporal vascular arcade was present without exudation (Fig. 1). An ultrasound B-scan was not performed due to the large exudation presented did not allow measurements properly. Autofluorescence did not show lipofuscin overlying the lesion (Fig. 1). Fluorescein angiography (FA) show a vascular network with small polypoidal structures producing a serous detachment of the retina in the macular region on the surface of the tumor (Fig. 1); indocyanine green angiography (ICG) showed 2 hypofluorescent lesions, correspondent with the alterations seen clinically, besides showing polypoidal lesions with minimal leakage in the late phases in the inferior lesion (Fig. 1). Optical coherence tomography (OCT) showed the presence of flat subfoveal fluid, serous pigmentary epithelial detachment, and intraretinal hiperrrefletive foci support with hard exudates (Fig. 1). A diagnosis of PCV associated with choroidal nevus was made. After receiving informed consent, PDT was administrated in the left eye. One month after PDT, increase in the area of exudation, hard exudates, and bleeding were observed (Fig. 2). It was decided to start treatment with 1 dose of intravitreal bevacizumab and evaluate response. One month after injection, improvement was observed: less area of exudation and hard exudates, no bleeding, and improvement in visual acuity. It was decided to inject 2 additional doses of intravitreal bevacizumab with 1-month interval. At the final visit, the patient referred improvement in her visual acuity, the BCVA on her left eye rose to 0.7. Fundus examination showed less hard exudates and no bleeding (Fig. 3). OCT showed no subretinal fluid, less intraretinal hiperreflective foci, and a homogeneous hiperreflective material accumulation under retinal pigmented epithelial (Fig. 3). Because bleeding was reduced significantly, an ultrasound B-scan was performed, which showed a minimally elevated dome-shaped, highly reflective choroidal lesion that measured 0.9 mm × 4.11 mm (Fig. 4). Follow-up FA and ICG were not performed because the patient was not able to tolerate such examinations when were performed initially. The patient was followed up during 6 months with no recurrence of exudation and final BCVA of 0.7.

## 3 Discussion

A choroidal nevus is rarely associated with PCV. This association can remain stable without exudation or symptoms.^[8,9]^ Focal laser photocoagulation^[5]^ and PDT^[6,7]^ have been successfully used to treat PCV associated with choroidal nevus. PDT is effective in closing the polypoidal lesions, but in certain cases, it cannot induce complete occlusion of them with recurrence of exudation and bleeding.^[10,11]^ We describe a patient with PCV associated with choroidal nevus which was successfully treated with 3 doses of intravitreal bevacizumab after 1 session of PDT. During the follow-up, no evidence of recurrence of exudation or bleeding, choroidal nevus growth, or malignant transformation was observed. To the best of our knowledge, this is the first report of PCV associated with a choroidal nevus that was successfully treated with intravitreal bevacizumab as an adjunctive treatment after PDT.

## 4 Conclusions

A choroidal nevus rarely is associated with PCV. Its behavior over time can be variable, with or without exudation, therefore, management can be performed with observation, laser, antiangiogenic agents, PDT, or a combination therapy. An appropriate option may be the combination of PDT plus antiangiogenic therapy.
